# APOE4 carriers have a unique cerebrospinal fluid and plasma proteome signature irrespective of cognitive diagnosis

**DOI:** 10.1002/alz70855_101734

**Published:** 2025-12-23

**Authors:** Artur Shvetcov, Heather M Wilkins, Jeffrey M. Burns, Russell H Swerdlow, Chad M Slawson, Caitlin A Finney, Global Neurodegeneration Proteomics Consortium

**Affiliations:** ^1^ Discipline of Psychiatry and Mental Health, University of New South Wales, Sydney, NSW, Australia; ^2^ Department of Psychological Medicine, Sydney Children's Hospitals Network, Sydney, NSW, Australia; ^3^ Translational Dementia Research Group, Westmead Institute for Medical Research, Westmead, NSW, Australia; ^4^ University of Kansas Alzheimer's Disease Research Center, Kansas City, KS, USA; ^5^ Department of Neurology, University of Kansas School of Medicine, Kansas City, KS, USA; ^6^ Department of Biochemistry and Molecular Biology, University of Kansas Medical Center, Kansas City, KS, USA; ^7^ Department of Cell Biology and Physiology, University of Kansas Medical Center, Kansas City, KS, USA; ^8^ University of Kansas Alzheimer's Disease Research Center, Fairway, KS, USA; ^9^ University of Kansas Cancer Center, University of Kansas Medical Center, Kansas City, KS, USA; ^10^ School of Medical Sciences, The University of Sydney, Sydney, NSW, Australia

## Abstract

**Background:**

Apolipoprotein E ε4 (APOE4) is the single biggest genetic risk factor for mild cognitive impairment (MCI) and sporadic Alzheimer's disease (AD). Despite this, little is known about the mechanisms underlying APOE4 carriers’ vulnerability to these diseases.

**Methods:**

We used the Global Neurodegeneration Proteomics Consortium dataset to obtain genetic and SomaScan 7k assay proteomic data from the plasma and cerebrospinal fluid (CSF) for 8965 non‐impaired controls (NI), 1283 AD, and 505 MCI patients. To identify proteins that were predictive of APOE4 (features), we first performed feature selection using mutual information followed by supervised machine learning using classification and regression trees (CART). Biological pathways associated with APOE4‐specific proteins were identified using functional network analyses.

**Results:**

Our CART models revealed that APOE4 carriers had a unique proteome signature across both the CSF and plasma. The proteome signature was independent of cognitive status and was found in NI, MCI, and AD patients. Functional enrichment analyses showed that these APOE4‐specific proteins were associated with apoptosis and inflammation as well as dysregulation in immune system DNA/RNA processes, mitochondrion organization, and glycolysis.

**Conclusion:**

Our findings demonstrate that APOE4 carriage defines both the CSF and plasma proteome irrespective of cognitive status. First, this suggests that APOE4 carriers have systemic biological alterations. Second, our findings suggest that the underlying biological changes in APOE4 carriers are essential but not sufficient for developing MCI and AD. It may be the case that these biological changes make APOE4 carriers more vulnerable to environmental insults, such as viral infections, and together these factors drive MCI and AD pathogenesis.

